# Supplementary material for: Predicting child development and school readiness, at age 5, for Aboriginal and non-Aboriginal children in Australia’s Northern Territory
Source: PLoS One. 2023 Dec 20;18(12):e0296051. doi: 10.1371/journal.pone.0296051 (PMC10732444; doi:10.1371/journal.pone.0296051)
Supplement: S1 File — (DOCX) [file pone.0296051.s001.docx]

**Supplementary information**

**Predicting child development and school readiness, at age 5, for Aboriginal and non-Aboriginal children in Australia’s Northern Territory**

Abel Fekadu Dadi^1*^, Vincent He^1^, Georgina Nutton2, Jiunn-Yih Su^1^, Steven Guthridge^1^

**^1^** Menzies School of Health Research, Charles Darwin University, Darwin, Northern Territory, Australia

^2^ College of Education, Charles Darwin University, Darwin, Northern Territory, Australia

^*^Corresponding author

Email: [abel.dadi@menzies.edu.au](mailto:abel.dadi@menzies.edu.au)

**Contents**

[Appendix I: Testing the model for clustering effect at school and teacher level 3](#_Toc117767574)

[Appendix II: Model for parameter reduction and confounding handling 6](#_Toc117767575)

[Appendix III: Potential predictive models 10](#_Toc117767576)

[Appendix IV. Selected competing models 27](#_Toc117767577)

[Model for Aboriginal children 27](#_Toc117767578)

[Model for non-Aboriginal children 29](#_Toc117767579)

[Appendix V: Distribution of prediction errors from PLS_SEM and linear regression models 31](#_Toc117767580)

# **Appendix I**: Testing the model for clustering effect at school and teacher level

**2^nd^ step** (testing model with both school and teacher level clustering effect over model accounting only school effect)

===== A model accounting for the two-level clustering effect is found to be better in explaining the clustering effect at all levels and used for selecting parameters for prediction model.

# **Appendix II**: Model for parameter reduction and confounding handling

We used three ways of variable selection, which we guided by the life span theoretical model in figure 1. We fitted the first model (Model I) by including all covariates suspected to predict the AEDC at the same time assuming that all covariates measured at different lifespans have equal contributions to the AEDC. The second (Model II) approach involved fitting separate regression models at each point of the developmental lifespan by only considering variables representing the context. The third (Model III) approach fitted a stepwise regression assuming that variables at each point of the developmental span interrelate and affect the childhood developmental outcomes (Table 1 to 3 in the supplementary information). We first tested the normality assumption of the AEDC scores for Aboriginal and non-Aboriginal children, and the AEDC for non-Aboriginal is not perfectly normally distributed. As such, we fitted a multivariable mixed-effect linear regression model for Aboriginal children and logistic regression with robust standard error for non-Aboriginal children. Furthermore, in every regression analysis, the three missing handling methods have been tested. In all models, a p-value from 0.05 to 0.08 was used to select potential candidate variables for the prediction model.

Table 1: Factors significantly associated with the AEDC score in Model I, using complete case analysis (CCA), missing indicator (MI), and multiply imputation (MICE), separately for the Aboriginal and non-Aboriginal populations. *(Note: all coefficients are significant at a p-value 0.05, * are marginally significant)*

|  | Type of risk factor | Model 1 | | | | | | | | |
| --- | --- | --- | --- | --- | --- | --- | --- | --- | --- | --- |
|  |  | CCA | | | MI | | | MICE | | |
|  |  | Aboriginal(N=345) | Non-aboriginal(N=486) | Total(N=831) | Aboriginal(N=981) | Non-aboriginal(N=782) | Total(N=1763) | Aboriginal (=1021) | Non-Aboriginal (=1194) | Total (=2215) |
|  | Remoteness |  |  |  |  |  | -1.29 | -1.268 |  | -1.29 |
|  | IRSD decile | 0.61 |  | 0.28 | 0.37* | 0.22 | 0.29 |  |  | .18 |
|  | Indigenous status |  |  | -2.41 |  |  | -3.05 |  |  | -2.60 |
|  | English as a second language | -2.36* |  | -2.63 | -4.18 | -1.20* | -3.11 | -3.33 | -1.24 | -2.75 |
|  | Mother’s occupation |  | 0.14 |  |  |  |  | 0.47* |  |  |
|  | Mother’s education |  |  |  | 0.37 |  | 0.31 |  |  | 0.31* |
|  | Age of mother at pregnancy |  | -0.06 | 0.08* |  | 0.09 | 0.09 |  | 0.08 | 0.07 |
|  | Number of pregnancies the mother had | -0.54* | 0.25 | -0.65 |  | -0.57 | -0.44 | -0.29* | -0.51 | -0.39 |
|  | Mother’s smoking in pregnancy | -1.58* | 0.50* | -1.26 |  |  |  |  |  |  |
|  | MC: pre-hypertension |  |  | 3.85* |  |  |  |  |  |  |
|  | MC: pre-diabetes | -9.04 |  | -7.46 |  |  |  |  |  |  |
|  | MC: cardiac disease |  |  |  |  |  | -1.54* |  | -4.89 | -1.70 |
|  | MC: renal disease |  |  |  | 3.88 |  | 3.06 | 3.71* |  | 2.61* |
|  | OC: gestational diabetes |  |  | -2.27* |  |  |  |  |  |  |
|  | OC: pre-eclampsia |  |  | -4.42 |  |  | -1.80* |  |  | -1.77* |
|  | OC: IUGR |  |  |  |  |  |  |  | -2.76* |  |
|  | LC: postpartum haemorrhage |  |  | -1.36 |  | -1.28 | -1.13 |  | -0.86* | -0.96 |
|  | Resuscitation |  | 0.39* |  |  |  |  |  |  | -0.55* |
|  | Twin birth |  | -2.93 |  | -3.84 |  |  | -3.88 |  |  |
|  | Gender of the newborn | 4.77 | -0.85 | 3.52 | 3.33 | 3.50 | 3.41 | 3.10 | 3.32 | 3.28 |
|  | Gestational age |  | -0.20 |  |  | 0.36* |  |  |  |  |
|  | Head circumference | 0.09* |  |  |  |  |  |  |  |  |
|  | Mother’s hospital stays before and after birth |  |  |  | -0.21 |  | -0.13 | -0.19 |  | -0.12 |
|  | Child number of hospitalization in the first three years |  | -0.17* |  |  | 0.50 | -0.51 |  |  |  |
|  | Number of notifications in the first three years |  |  |  |  | -0.81* |  |  | -0.80* | -0.42 |
|  | Number of accidental injuries |  |  |  |  | -0.77 |  |  | -0.62 |  |
|  | Gastroenteritis in children |  |  | -2.56 | -1.63 | -3.17 | -1.95 | -1.36 | -2.44 | -1.59 |
|  | Acute lower respiratory infection |  |  |  |  | -1.89* |  |  |  |  |
|  | Preschool attendance | 6.23 |  | 4.87 |  |  | -0.01 | 3.59 | 1.73* | 2.94 |
|  | AIC | 2486.281 | 3205.75 | 5673.461 | 6946.96 | 5112.731 | 12070.52 |  |  |  |
|  | BIC | 2659.241 | 3385.757 | 5881.257 | 7157.169 | 5313.191 | 12311.41 |  |  |  |

Table 2: Factors significantly associated with the AEDC score in Model II using complete case analysis (CCA), missing indicator (MI), and multiply imputation (MICE), separately for the Aboriginal and non-Aboriginal populations. *(Note: all coefficients are significant at a p-value 0.05, * are marginally significant)*

|  | Type of risk factor | Model 2 | | | | | | | | |
| --- | --- | --- | --- | --- | --- | --- | --- | --- | --- | --- |
|  |  | CCA | | | MI | | | MICE | | |
|  |  | Aboriginal(N=925) | Non-aboriginal(N=1122) | Total(N=1462) | Aboriginal(N=1030) | Non-aboriginal(N=1202) | Total(N=2232) | Aboriginal (=1025) | Non-aboriginal (=1201) | Total (=1651) |
|  | Remoteness |  |  | -1.09 | -2.12 |  | -1.54 | -1.95 |  | -1.86 |
|  | IRSD decile | 0.51 |  | 0.26 |  | 0.13* | 0.21 |  |  |  |
|  | Indigenous status |  |  | -2.81 |  |  | -2.93 |  |  | -2.67 |
|  | English as a second language | -4.56 |  | -2.47 | -4.51 | -1.33 | -3.10 | -3.74 | -1.29 | -2.66 |
|  | Mother’s occupation |  |  |  |  |  |  | 0.64 |  |  |
|  | Mother’s education |  |  | 0.67 |  | 0.33 | 0.32 |  |  |  |
|  | Age of mother at pregnancy |  | -0.04 | -2.67 |  | 0.08 | 0.08 |  | 0.10 | 0.14 |
|  | Number of pregnancies the mother had |  | 0.14 |  | -0.24* | -0.46 | -0.43 |  | -0.54 | -0.63 |
|  | Mother’s smoking in pregnancy |  |  |  |  |  |  |  |  | -0.89 |
|  | MC: pre-diabetes |  |  |  |  |  |  |  |  | -3.30* |
|  | MC: cardiac disease |  |  |  |  | -4.81 | -1.77 |  |  |  |
|  | MC: renal disease |  |  |  | 3.95 |  | 2.72* |  |  |  |
|  | OC: gestational diabetes |  |  |  |  |  |  |  |  | -1.34* |
|  | OC: UTI |  |  | -2.24* |  |  |  |  |  |  |
|  | OC: IUGR |  |  |  |  |  |  |  | -3.80 |  |
|  | LC: postpartum hemorrhage |  | 0.39* | -1.25 | -1.22* | -0.99 | -1.05 | -1.14* |  | -1.54 |
|  | Resuscitation |  |  | -0.63* |  |  |  |  |  |  |
|  | Twin birth | -3.85 |  |  | -3.22 |  |  | -3.89 |  |  |
|  | Gender of the newborn | 3.33 | -0.83 | 3.57 | 3.38 | 3.22 | 3.31 | 3.40 | 3.28 | 3.23 |
|  | Birth weight |  |  |  |  |  |  |  |  | 1.08 |
|  | Gestational age |  | -0.11 |  |  | 0.29 |  |  |  |  |
|  | Mother’s hospital stays before and after birth | -0.20 |  |  | -0.23 |  | -0.17 | -0.19 |  | -0.11* |
|  | Child number of hospitalization in the first three years |  |  | -0.25* |  |  |  |  |  |  |
|  | Number of notifications in the first three years |  | 0.26* |  |  | -0.91 | -0.46 |  | -0.92 |  |
|  | CP older sibling | -1.31 |  |  |  |  |  | -1.07* |  |  |
|  | Number of accidental injuries |  |  |  |  | -0.53 |  |  | -0.48* |  |
|  | Gastroenteritis in children |  |  |  | -1.66 | -2.27 | -1.83 |  | -2.25 | -1.51 |
|  | Preschool attendance | 3.46 |  | 3.79 | -0.01* |  | -0.01 | 3.42 |  | 3.19 |
|  | AIC | 6496.274 | 7165.304 | 9818.914 | 7244.662 | 7671.13 | 15002.16 |  |  |  |
|  | BIC | 6592.87 | 7275.808 | 9961.678 | 7348.346 | 7788.24 | 15144.93 |  |  |  |

Table 3: Factors significantly associated with the AEDC score in Model III using complete case analysis (CCA), missing indicator (MI), and multiply imputation (MICE), separately for the Aboriginal and non-Aboriginal population. *(Note: all coefficients are significant at p-value 0.05, * are marginally significant)*

|  | Type of risk factor | Model 3 | | | | | | | | |
| --- | --- | --- | --- | --- | --- | --- | --- | --- | --- | --- |
|  |  | CCA | | | MI | | | MICE | | |
|  |  | Aboriginal(N=925) | Non-aboriginal(N=883) | Total(N=1078) | Aboriginal(N=1030) | Non-aboriginal(N=1201) | Total(N=2232) | Aboriginal (=708) | Non-aboriginal (=940) | Total (=1647) |
|  | Remoteness |  |  |  | -1.27* |  | -1.52 | -1.96 |  | -1.46 |
|  | IRSD decile | 0.62 | -0.06 | 0.23 | 0.37 |  | 0.21 |  |  |  |
|  | Indigenous status |  |  | -2.57 |  |  | -2.9 |  |  | -2.50 |
|  | English as a second language | -2.82 |  | -2.35 | -4.24 | -1.35 | -3.06 | -2.72 | -1.55 | -2.41 |
|  | Mother’s occupation |  |  |  |  |  |  | 0.51* |  |  |
|  | Mother’s education |  |  | 0.43* |  | 0.34 | 0.33 |  |  |  |
|  | Age of mother at pregnancy |  | -0.06 |  |  | 0.07 | 0.08 |  | 0.09 | 0.12 |
|  | Number of pregnancies the mother had |  | 0.19 | -0.40 | -0.22* | -0.45 | -0.43 |  | -0.60 | -0.61 |
|  | Mother’s smoking in pregnancy | -1.88 |  | -1.10 |  |  |  | -1.17* |  | -0.75* |
|  | MC: pre-diabetes |  |  | -5.56 |  |  |  |  |  | -3.31* |
|  | MC: cardiac disease |  |  |  |  | -4.88 | -1.66 |  |  |  |
|  | MC: renal disease |  |  |  | 4.54 |  | 2.99 |  |  |  |
|  | OC: pre-eclampsia |  |  |  |  |  | -1.57 |  |  |  |
|  | OC: UTI |  |  | -2.88* |  |  |  |  |  | -1.97 |
|  | OC: IUGR |  |  |  |  | -3.42 |  |  | -3.63 |  |
|  | LC: postpartum hemorrhage |  |  | -1.02* | -1.26 | -0.96* | -1.04 | -2.10 |  | -1.44 |
|  | Twin birth |  | -1.67 |  |  |  |  |  |  |  |
|  | Gender of the newborn | 4.08 | -1.0 | 3.38 | 3.34 | 3.16 | 3.30 | 2.93 | 3.03 | 3.19 |
|  | Birth weight |  |  |  |  |  |  |  |  | 1.03 |
|  | Gestational age |  | -0.14 |  |  | 0.23 |  |  | 0.30 |  |
|  | Mother’s hospital stays before and after birth |  |  |  | -0.24 |  | -0.14 | -0.25 |  | -0.14 |
|  | Child number of hospitalization in the first three years | -0.44* |  | -0.34* |  |  |  |  |  |  |
|  | Number of notifications in the first three years |  |  |  |  | -0.77 | -0.38 |  |  | -0.32* |
|  | CP older sibling |  |  |  | -1.02* |  |  |  |  |  |
|  | Number of accidental injuries |  |  |  |  |  |  |  | -0.67 |  |
|  | Gastroenteritis in children |  |  | -2.38 | -2.24 | -2.32 | -1.84 |  | -2.38 | -1.80 |
|  | Acute lower respiratory infection |  |  |  |  |  |  |  |  |  |
|  | Preschool attendance | 6.23 |  | 4.69 | -0.01 |  | -0.01 | 4.58 |  | 3.21 |
|  | AIC | 2627.667 | 5667.359 | 7252.878 | 7243.24 | 7655.9 | 15001.09 |  |  |  |
|  | BIC | 2690.24 | 5763.025 | 7377.45 | 7337.049 | 7747.536 | 15155.28 |  |  |  |

# **Appendix III: Potential predictive models**

**1.1. Model fitting for Aboriginal children**

**Model 1**: The model is fitted using all potential covariates based on a proposed theoretical model and excluding not important path coefficients stepwise

**In sample model fit**

R^2^=0.274

Q^2^=0.105

NFI=0.86

SRMR=0.07

**Out of sample prediction power**

Q^2^predict= 0.242

RMSE= 0.874

MAE= 0.721

AIC= -214.489

BIC= -200.932

**Prediction power assessment**

| Indicators | PLS-SEM | | LM | | PLSEM-LM (RMSE) | PLSEM-LM (MAE) |
| --- | --- | --- | --- | --- | --- | --- |
|  | RMSE | MAE | RMSE | MAE |  |  |
| PHYS | 1.773 | 1.461 | 1.769 | 1.448 | 0.04 | 0.13 |
| SOC | 2.196 | 1.870 | 2.196 | 1.871 | 0 | -0.01 |
| EMOT | 1.938 | 1.584 | 1.937 | 1.587 | 0.01 | -0.03 |
| LANGCOG | 2.364 | 1.960 | 2.365 | 1.959 | -0.01 | 0.01 |
| COMGEN | 2.836 | 2.407 | 2.833 | 2.393 | 0.03 | 0.14 |

**Model 2**: The model is fitted using all significant variables from complete case analysis in Model I as a composite latent variable

**In sample model fit**

R^2^=0.221

Q^2^=0.111

NFI=0.96

SRMR=0.047

**Out of sample prediction power**

Q^2^predict= 0.137

RMSE= 0.932

MAE= 0.766

AIC= -142.22

BIC= -120.184

**Prediction power assessment**

| Indicators | PLS-SEM | | LM | | PLSEM-LM (RMSE) | PLSEM-LM (MAE) |
| --- | --- | --- | --- | --- | --- | --- |
|  | RMSE | MAE | RMSE | MAE |  |  |
| PHYS | 1.785 | 1.430 | 1.786 | 1.431 | -0.001 | -0.01 |
| SOC | 2.161 | 1.841 | 2.161 | 1.844 | 0 | -0.003 |
| EMOT | 1.926 | 1.588 | 1.926 | 1.592 | 0 | -0.004 |
| LANGCOG | 2.395 | 2.015 | 2.394 | 2.013 | 0.01 | 0.002 |
| COMGEN | 2.915 | 2.492 | 2.916 | 2.494 | -0.001 | -0.002 |

**Model 3:** The model is fitted by taking all significant variables from the complete case analysis in Model I as single latent variables

**Model fit**

R^2^=0.233

Q^2^=0.127

NFI=0.969

SRMR=0.032

**Predictive adequacy**

Q^2^predict= 0.145

RMSE= 0.926

MAE= 0.773

AIC= -144.017

BIC= -104.355

**Prediction power assessment**

| Indicators | PLS-SEM | | LM | | PLSEM-LM (RMSE) | PLSEM-LM (MAE) |
| --- | --- | --- | --- | --- | --- | --- |
|  | RMSE | MAE | RMSE | MAE |  |  |
| PHYS | 1.776 | 1.417 | 1.777 | 1.421 | -0.001 | -0.004 |
| SOC | 2.124 | 1.815 | 2.103 | 1.802 | 0.021 | 0.013 |
| EMOT | 1.887 | 1.554 | 1.858 | 1.533 | 0.028 | 0.021 |
| LANGCOG | 2.393 | 2.015 | 2.391 | 2.013 | 0.002 | 0.002 |
| COMGEN | 2.903 | 2.488 | 2.910 | 2.496 | -0.007 | -0.008 |

**Model 4: The model is re-fitted after excluding non-significant paths from model 3**

**In sample model fit**

R^2^=0.263

Q^2^=0.138

NFI=0.983

SRMR=0.041

**Out of sample prediction power**

Q^2^predict= 0.170

RMSE= 0.913

MAE= 0.759

**Prediction power assessment**

| Indicators | PLS-SEM | | LM | | PLSEM-LM (RMSE) | PLSEM-LM (MAE) |
| --- | --- | --- | --- | --- | --- | --- |
|  | RMSE | MAE | RMSE | MAE |  |  |
| PHYS | 1.746 | 1.403 | 1.740 | 1.401 | 0.006 | 0.002 |
| SOC | 2.177 | 1.865 | 2.140 | 1.817 | 0.037 | 0.048 |
| EMOT | 1.918 | 1.567 | 1.887 | 1.539 | 0.031 | 0.028 |
| LANGCOG | 2.404 | 2.008 | 2.404 | 2.007 | 0 | 0.001 |
| COMGEN | 2.903 | 2.465 | 2.902 | 2.470 | 0.001 | 0.005 |

**Model 5**: Revised path model after excluding non-important indicator (Physical domain) based on loading weights

**In sample model fit**

R^2^=0.263

Q^2^=0.149

NFI=0.982

SRMR=0.043

**Out of sample prediction power**

Q^2^predict= 0.170

RMSE= 0.915

MAE= 0.761

**Prediction power assessment**

| Indicators | PLS-SEM | | LM | | PLSEM-LM (RMSE) | PLSEM-LM (MAE) |
| --- | --- | --- | --- | --- | --- | --- |
|  | RMSE | MAE | RMSE | MAE |  |  |
| PHYS | 1.746 | 1.403 | 1.740 | 1.401 | 0.006 | 0.023 |
| SOC | 2.180 | 1.867 | 2.156 | 1.838 | 0.024 | 0.029 |
| EMOT | 1.921 | 1.570 | 1.894 | 1.546 | 0.027 | 0.024 |
| LANGCOG | 2.409 | 2.011 | 2.410 | 2.011 | -0.001 | 0 |
| COMGEN | 2.905 | 2.468 | 2.906 | 2.475 | -0.001 | -0.007 |

1.2 Models fitting for non-Aboriginal children

**Model 6**: Fitted based on the original life span theoretical model and non-important variables were reduced step by step

**Model fit**

R^2^=0.117

Q^2^=0.052

NFI=0.93

SRMR=0.037

**Predictive adequacy**

Q^2^predict= 0.066

RMSE= 0.969

MAE= 0.710

AIC= -145.743

BIC= -125.310

**Prediction power assessment**

| Indicators | PLS-SEM | | LM | | PLSEM-LM (RMSE) | PLSEM-LM (MAE) |
| --- | --- | --- | --- | --- | --- | --- |
|  | RMSE | MAE | RMSE | MAE |  |  |
| PHYS | 1.332 | 1.041 | 1.334 | 1.042 | -0.002 | -0.001 |
| SOC | 1.726 | 1.394 | 1.724 | 1.395 | 0.002 | -0.001 |
| EMOT | 1.637 | 1.277 | 1.632 | 1.281 | 0.005 | -0.004 |
| LANGCOG | 1.452 | 1.074 | 1.453 | 1.072 | -0.001 | 0.002 |
| COMGEN | 2.029 | 1.630 | 2.031 | 1.627 | -0.002 | 0.003 |

**Model 7:** Fitted based on variables significant in CCA in model 1 as a latent construct

**Model fit**

R^2^=0.120

Q^2^=0.058

NFI=0.959

SRMR=0.034

**Predictive adequacy**

Q^2^predict= 0.075

RMSE= 0.965

MAE= 0.720

AIC= -90.66

BIC= -67.37

**Prediction power assessment**

| Indicators | PLS-SEM | | LM | | PLSEM-LM (RMSE) | PLSEM-LM (MAE) |
| --- | --- | --- | --- | --- | --- | --- |
|  | RMSE | MAE | RMSE | MAE |  |  |
| PHYS | 1.363 | 1.056 | 1.366 | 1.063 | -0.003 | - 0.007 |
| SOC | 1.764 | 1.418 | 1.749 | 1.406 | 0.015 | 0.012 |
| EMOT | 1.652 | 1.273 | 1.622 | 1.250 | 0.030 | 0.023 |
| LANGCOG | 1.519 | 1.105 | 1.511 | 1.102 | 0.008 | 0.003 |
| COMGEN | 2.106 | 1.699 | 2.106 | 1.686 | 0 | 0.013 |

**Model 8:** The model is fitted by taking all significant variables from complete case analysis in Model I as single latent variables

**Model fit**

R^2^=0.10

Q^2^=0.055

NFI=0.989

SRMR=0.020

**Predictive adequacy**

Q^2^predict= 0.069

RMSE= 0.970

MAE= 0.747

AIC= -118.116

BIC= -97.749

**Prediction power assessment**

| Indicators | PLS-SEM | | LM | | PLSEM-LM (RMSE) | PLSEM-LM (MAE) |
| --- | --- | --- | --- | --- | --- | --- |
|  | RMSE | MAE | RMSE | MAE |  |  |
| EMO | 1.59 | 1.242 | 1.588 | 1.24 | 0.002 | 0.002 |
| PHYS | 1.334 | 1.034 | 1.334 | 1.036 | 0 | -0.002 |
| SOC | 1.702 | 1.372 | 1.703 | 1.371 | -0.001 | 0.001 |
| COMGEN | 2.022 | 1.607 | 2.023 | 1.606 | -0.001 | 0.001 |
| LANGCOG | 1.483 | 1.091 | 1.482 | 1.095 | 0.001 | -0.004 |

**Model 9: Fitted by controlling confounders in PLS-SEM**

**Model fit**

R^2^=0.105

Q^2^=0.04

NFI=0.92

SRMR=0.05

**Predictive adequacy**

Q^2^predict= 0.04

RMSE= 0.98

MAE= 0.74

AIC= -128.24

BIC= -107.81

**Prediction power assessment**

| Indicators | PLS-SEM | | LM | | PLSEM-LM (RMSE) | PLSEM-LM (MAE) |
| --- | --- | --- | --- | --- | --- | --- |
|  | RMSE | MAE | RMSE | MAE |  |  |
| PHYS | 1.335 | 1.044 | 1.336 | 1.044 | -0.001 | 0 |
| SOC | 1.730 | 1.403 | 1.726 | 1.399 | 0.004 | 0.004 |
| EMOT | 1.634 | 1.277 | 1.632 | 1.280 | 0.002 | -0.003 |
| LANGCOG | 1.453 | 1.075 | 1.451 | 1.071 | 0.002 | 0.004 |
| COMGEN | 2.030 | 1.622 | 2.032 | 1.629 | -0.002 | -0.007 |

**II. Predictive model from Model II complete case analysis**

**2.1. Model fitting for Aboriginal children**

Model 10: Fitted using significant variables as composite latent variables

**Model fit**

R^2^=0.273

Q^2^=0.132

NFI=0.97

SRMR=0.05

**Predictive adequacy**

Q^2^predict= 0.158

RMSE= 0.920

MAE= 0.764

AIC= -278.60

BIC= -268.282

**Prediction power assessment**

| Indicators | PLS-SEM | | LM | | PLSEM-LM (RMSE) | PLSEM-LM (MAE) |
| --- | --- | --- | --- | --- | --- | --- |
|  | RMSE | MAE | RMSE | MAE |  |  |
| PHYS | 1.759 | 1.419 | 1.760 | 1.419 | -0.001 | 0 |
| SOC | 2.211 | 1.894 | 2.211 | 1.896 | 0 | -0.002 |
| EMOT | 1.953 | 1.598 | 1.953 | 1.600 | 0 | -0.002 |
| LANGCOG | 2.420 | 2.022 | 2.420 | 2.021 | 0 | 0.001 |
| COMGEN | 2.923 | 2.483 | 2.923 | 2.485 | 0 | -0.002 |

Model 11: Significant variables were fitted independently (all paths are significant)

**Model fit**

R^2^=0.296

Q^2^=0.155

NFI=0.98

SRMR=0.033

**Predictive adequacy**

Q^2^predict= 0.188

RMSE= 0.903

MAE= 0.751

AIC= -309.686

BIC= -271.047

**Prediction power assessment**

| Indicators | PLS-SEM | | LM | | PLSEM-LM (RMSE) | PLSEM-LM (MAE) |
| --- | --- | --- | --- | --- | --- | --- |
|  | RMSE | MAE | RMSE | MAE |  |  |
| PHYS | 1.736 | 1.398 | 1.731 | 1.396 | 0.005 | 0.002 |
| SOC | 2.168 | 1.859 | 2.144 | 1.830 | 0.024 | 0.029 |
| EMOT | 1.910 | 1.562 | 1.883 | 1.540 | 0.027 | 0.022 |
| LANGCOG | 2.379 | 1.991 | 2.378 | 1.990 | 0.001 | 0.001 |
| COMGEN | 2.892 | 2.458 | 2.895 | 2.465 | -0.003 | -0.007 |

**2.2. Model fitting for non-Aboriginal children**

**Model 12**: significant variables were fitted as a composite latent construct

**Model fit**

R^2^=0.12

Q^2^=0.065

NFI=0.948

SRMR=0.034

**Predictive adequacy**

Q^2^predict= 0.026

RMSE= 0.992

MAE= 0.771

AIC= -148.552

BIC= -128.119

**Prediction power assessment**

| Indicators | RMSE |  |  | MAE |  |  |
| --- | --- | --- | --- | --- | --- | --- |
|  | PLS-SEM | LM | PLSEM-LM | PLS-SEM | LM | PLSEM-LM |
| EMOT | 1.646 | 1.644 | 0.002 | 1.29 | 1.292 | -0.002 |
| PHYS | 1.341 | 1.342 | -0.001 | 1.052 | 1.052 | 0 |
| SOC | 1.735 | 1.737 | -0.002 | 1.407 | 1.409 | -0.002 |
| COMGEN | 2.043 | 2.044 | -0.001 | 1.648 | 1.646 | 0.002 |
| LANGCOG | 1.478 | 1.477 | 0.001 | 1.102 | 1.097 | 0.005 |

**Model 13**: Fitted using significant predictors in model 2 as independent latent factors

**Model fit**

R^2^=0.121

Q^2^=0.066

NFI=0.981

SRMR=0.020

**Predictive adequacy**

Q^2^predict= 0.079

RMSE= 0.965

MAE= 0.747

AIC= -141.869

BIC= -106.226

**Prediction power assessment**

| Indicators | RMSE |  |  | MAE |  |  |
| --- | --- | --- | --- | --- | --- | --- |
|  | PLS-SEM | LM | PLSEM-LM | PLS-SEM | LM | PLSEM-LM |
| EMOT | 1.591 | 1.586 | 0.005 | 1.235 | 1.234 | 0.001 |
| PHYS | 1.322 | 1.323 | -0.001 | 1.025 | 1.027 | -0.002 |
| SOC | 1.693 | 1.694 | -0.001 | 1.362 | 1.36 | 0.002 |
| COMGEN | 2.007 | 2.005 | 0.002 | 1.593 | 1.585 | 0.008 |
| LANGCOG | 1.46 | 1.456 | 0.004 | 1.075 | 1.073 | 0.002 |

**III. Predictive model from Model III complete case analysis**

**3.1. Model fitting for Aboriginal children**

**Model 14**: Fitting using significant factors as composite factors (latent construct)

**Model fit**

R^2^=0.248

Q^2^=0.122

NFI=0.964

SRMR=0.054

**Predictive adequacy**

Q^2^predict= 0.170

RMSE= 0.914

MAE= 0.757

AIC= -173.124

BIC= -155.135

**Prediction power assessment**

| Indicators | PLS-SEM | | LM | | PLSEM-LM (RMSE) | PLSEM-LM (MAE) |
| --- | --- | --- | --- | --- | --- | --- |
|  | RMSE | MAE | RMSE | MAE |  |  |
| PHYS | 1.769 | 1.411 | 1.767 | 1.408 | 0.002 | 0.003 |
| SOC | 2.142 | 1.828 | 2.114 | 1.808 | 0.028 | 0.02 |
| EMOT | 1.910 | 1.573 | 1.878 | 1.540 | 0.032 | 0.033 |
| LANGCOG | 2.368 | 1.980 | 2.368 | 1.979 | 0 | 0.001 |
| COMGEN | 2.885 | 2.460 | 2.886 | 2.467 | -0.001 | -0.007 |

**Model 15**: Fitting risk factors independently as a latent variable (all paths and outer weights are significant except the physical domain)

**Model fit**

R^2^=0.256

Q^2^=0.122

NFI=0.974

SRMR=0.038

**Predictive adequacy**

Q^2^predict= 0.170

RMSE= 0.912

MAE= 0.757

AIC= -175.49

BIC= -148.807

**Prediction power assessment**

| Indicators | PLS-SEM | | LM | | PLSEM-LM (RMSE) | PLSEM-LM (MAE) |
| --- | --- | --- | --- | --- | --- | --- |
|  | RMSE | MAE | RMSE | MAE |  |  |
| PHYS | 1.768 | 1.409 | 1.765 | 1.408 | 0.003 | 0.001 |
| SOC | 2.139 | 1.823 | 2.113 | 1.807 | 0.026 | 0.016 |
| EMOT | 1.907 | 1.568 | 1.878 | 1.539 | 0.029 | 0.029 |
| LANGCOG | 2.367 | 1.979 | 2.367 | 1.978 | 0 | 0.001 |
| COMGEN | 2.885 | 2.460 | 2.885 | 2.468 | 0 | -0.008 |

**3.2. Model fitting for non-Aboriginal children**

**Model 16**.: Fitting using significant factors as composite factors (latent construct)

**Model fit**

R^2^=0.121

Q^2^=0.063

NFI=0.981

SRMR=0.024

**Predictive adequacy**

Q^2^predict= 0.035

RMSE= 0.987

MAE= 0.765

AIC= -150.340

BIC= -135.065

**Prediction power assessment**

| Indicators | RMSE |  |  | MAE |  |  |
| --- | --- | --- | --- | --- | --- | --- |
|  | PLS-SEM | LM | PLSEM-LM | PLS-SEM | LM | PLSEM-LM |
| EMOT | 1.641 | 1.639 | 0.002 | 1.287 | 1.288 | -0.001 |
| PHYS | 1.339 | 1.34 | -0.001 | 1.047 | 1.048 | -0.001 |
| SOC | 1.733 | 1.734 | -0.001 | 1.407 | 1.408 | -0.001 |
| COMGEN | 2.043 | 2.044 | -0.001 | 1.644 | 1.643 | 0.001 |
| LANGCOG | 1.474 | 1.471 | 0.003 | 1.097 | 1.092 | 0.005 |

**Model 17**: Fitting using significant factors as independent latent constructs

**Model fit**

R^2^=0.123

Q^2^=0.065

NFI=0.986

SRMR=0.018

**Predictive adequacy**

Q^2^predict= 0.089

RMSE= 0.958

MAE= 0.730

AIC= -144.175

BIC= -108.533

**Prediction power assessment**

| Indicators | RMSE |  |  | MAE |  |  |
| --- | --- | --- | --- | --- | --- | --- |
|  | PLS-SEM | LM | PLSEM-LM | PLS-SEM | LM | PLSEM-LM |
| EMOT | 1.59 | 1.583 | 0.007 | 1.239 | 1.236 | 0.003 |
| PHYS | 1.323 | 1.325 | -0.002 | 1.025 | 1.028 | -0.003 |
| SOC | 1.695 | 1.694 | 0.001 | 1.365 | 1.363 | 0.002 |
| COMGEN | 2.018 | 2.019 | -0.001 | 1.605 | 1.601 | 0.004 |
| LANGCOG | 1.459 | 1.453 | 0.006 | 1.071 | 1.07 | 0.001 |

# **Appendix IV. Selected competing models**

## **Model for Aboriginal children**

**Model 2**: The model is fitted using all significant variables from the complete case analysis in Model I as a composite latent variable

**In sample model fit**

R^2^=0.221

Q^2^=0.111

NFI=0.96

SRMR=0.047

**Out of sample prediction power**

Q^2^predict= 0.137

RMSE= 0.932

MAE= 0.766

AIC= -142.22

BIC= -120.184

**Prediction power assessment**

| Indicators | PLS-SEM | | LM | | PLSEM-LM (RMSE) | PLSEM-LM (MAE) |
| --- | --- | --- | --- | --- | --- | --- |
|  | RMSE | MAE | RMSE | MAE |  |  |
| PHYS | 1.785 | 1.430 | 1.786 | 1.431 | -0.001 | -0.01 |
| SOC | 2.161 | 1.841 | 2.161 | 1.844 | 0 | -0.003 |
| EMOT | 1.926 | 1.588 | 1.926 | 1.592 | 0 | -0.004 |
| LANGCOG | 2.395 | 2.015 | 2.394 | 2.013 | 0.01 | 0.002 |
| COMGEN | 2.915 | 2.492 | 2.916 | 2.494 | -0.001 | -0.002 |

Based on the guideline the model has **moderate to high predictive** power

Model 10: Fitted using a significant variable as composite latent variables

**Model fit**

R^2^=0.273

Q^2^=0.132

NFI=0.97

SRMR=0.05

**Predictive adequacy**

Q^2^predict= 0.158

RMSE= 0.920

MAE= 0.764

AIC= -278.60

BIC= -268.282

**Prediction power assessment**

| Indicators | PLS-SEM | | LM | | PLSEM-LM (RMSE) | PLSEM-LM (MAE) |
| --- | --- | --- | --- | --- | --- | --- |
|  | RMSE | MAE | RMSE | MAE |  |  |
| PHYS | 1.759 | 1.419 | 1.760 | 1.419 | -0.001 | 0 |
| SOC | 2.211 | 1.894 | 2.211 | 1.896 | 0 | -0.002 |
| EMOT | 1.953 | 1.598 | 1.953 | 1.600 | 0 | -0.002 |
| LANGCOG | 2.420 | 2.022 | 2.420 | 2.021 | 0 | 0.001 |
| COMGEN | 2.923 | 2.483 | 2.923 | 2.485 | 0 | -0.002 |

Based on the guideline the model has **low to moderate predictive power**

## **Model for non-Aboriginal children**

Model 6: Fitted based on the original life span theoretical model and non-important variables were reduced step by step

**Model fit**

R^2^=0.117

Q^2^=0.052

NFI=0.93

SRMR=0.037

**Predictive adequacy**

Q^2^predict= 0.066

RMSE= 0.969

MAE= 0.710

AIC= -145.743

BIC= -125.310

**Prediction power assessment**

| Indicators | PLS-SEM | | LM | | PLSEM-LM (RMSE) | PLSEM-LM (MAE) |
| --- | --- | --- | --- | --- | --- | --- |
|  | RMSE | MAE | RMSE | MAE |  |  |
| PHYS | 1.332 | 1.041 | 1.334 | 1.042 | -0.002 | -0.001 |
| SOC | 1.726 | 1.394 | 1.724 | 1.395 | 0.002 | -0.001 |
| EMOT | 1.637 | 1.277 | 1.632 | 1.281 | 0.005 | -0.004 |
| LANGCOG | 1.452 | 1.074 | 1.453 | 1.072 | -0.001 | 0.002 |
| COMGEN | 2.029 | 1.630 | 2.031 | 1.627 | -0.002 | 0.003 |

Based on the guideline the model has **low to moderate** predictive power

**Model 16**.: Fitting using significant factors as composite factors (latent construct)

**Model fit**

R^2^=0.121

Q^2^=0.063

NFI=0.981

SRMR=0.024

**Predictive adequacy**

Q^2^predict= 0.035

RMSE= 0.987

MAE= 0.765

AIC= -150.340

BIC= -135.065

**Prediction power assessment**

| Indicators | RMSE |  |  | MAE |  |  |
| --- | --- | --- | --- | --- | --- | --- |
|  | PLS-SEM | LM | PLSEM-LM | PLS-SEM | LM | PLSEM-LM |
| EMOT | 1.641 | 1.639 | 0.002 | 1.287 | 1.288 | -0.001 |
| PHYS | 1.339 | 1.34 | -0.001 | 1.047 | 1.048 | -0.001 |
| SOC | 1.733 | 1.734 | -0.001 | 1.407 | 1.408 | -0.001 |
| COMGEN | 2.043 | 2.044 | -0.001 | 1.644 | 1.643 | 0.001 |
| LANGCOG | 1.474 | 1.471 | 0.003 | 1.097 | 1.092 | 0.005 |

Based on the guideline the model has **low to less moderate predictive power**

# **Appendix V**: Distribution of prediction errors from PLS_SEM and linear regression models

**PHYS**

**SOC**


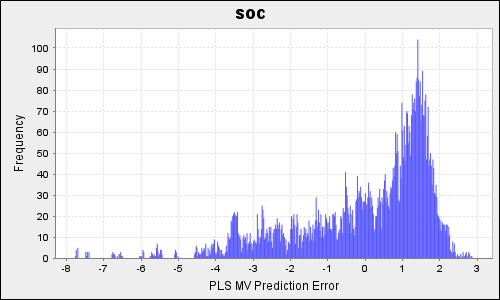

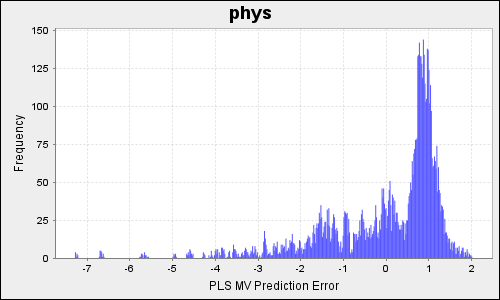

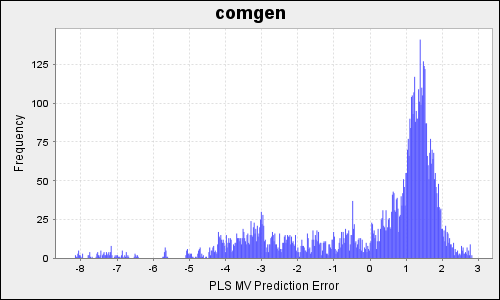

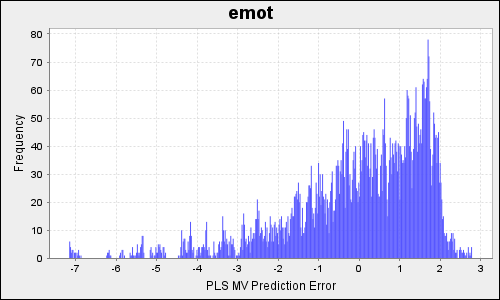

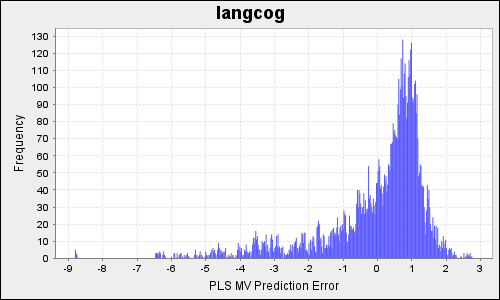

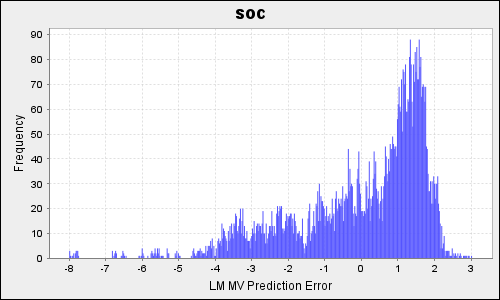

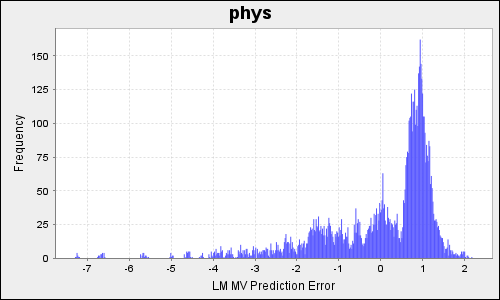

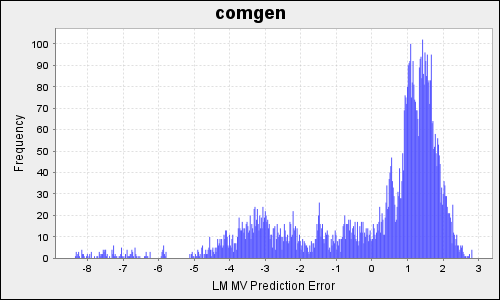

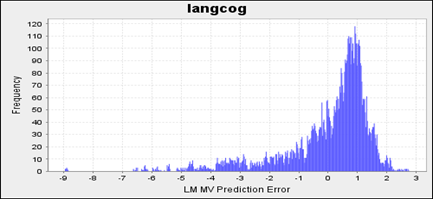

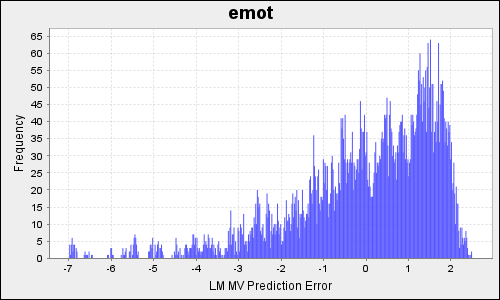


**EMOT**

**LANGCOG**

**COMGEN**

**PHYS**

**SOC**

**LANGCOG**

**EMOT**

**COMGEN**

Distribution of Prediction errors from PLS-SEM Distribution of Prediction errors from LM


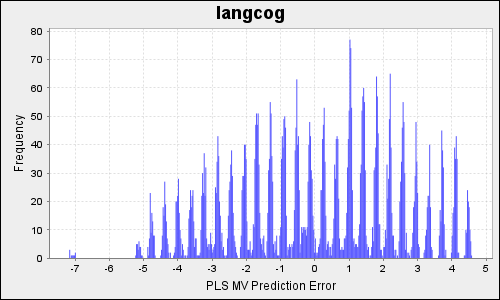

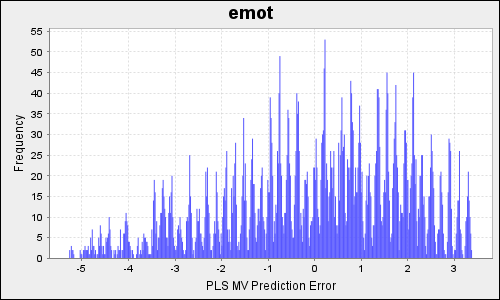

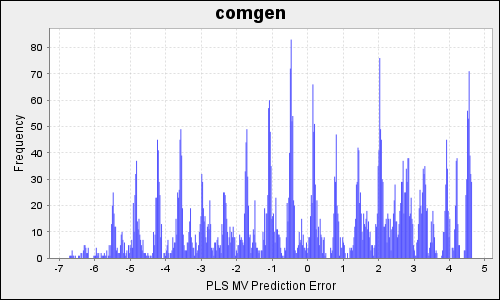

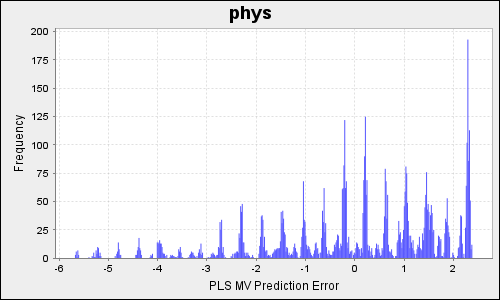

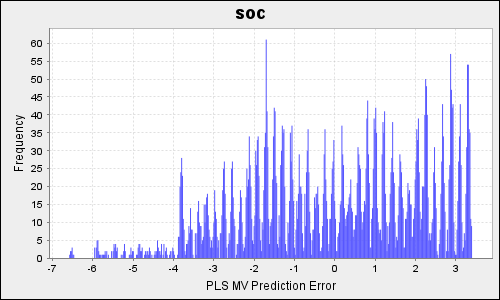

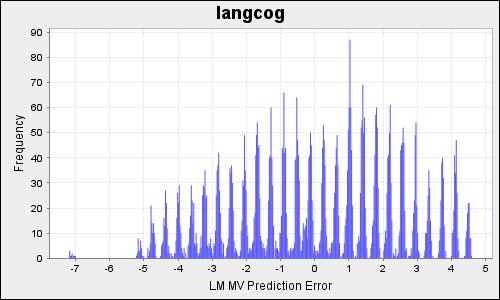

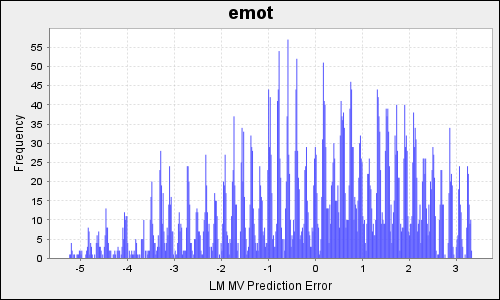

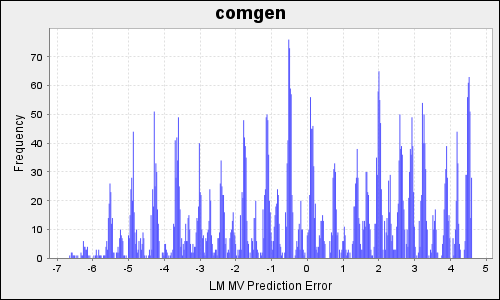

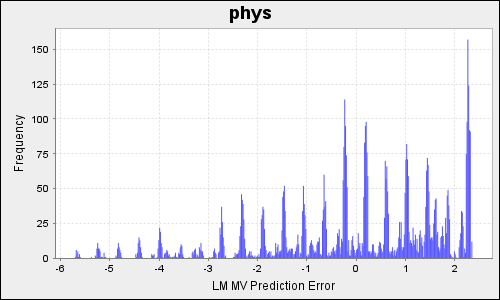

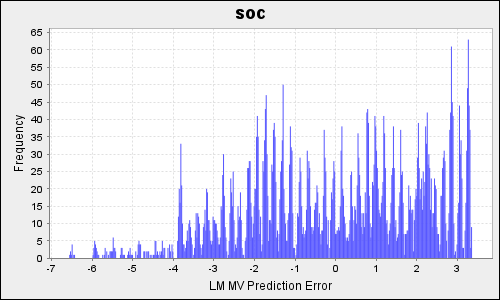


**SOC**

**SOC**

**PHYS**

**PHYS**

**COMGEN**

**COMGEN**

**EMOT**

**EMOT**

**LANGCOG**

**LANGCOG**

Distribution of prediction errors from PLS-SEM Distribution of prediction errors from LM
