# Supplementary material for: Predicting child development and school readiness, at age 5, for Aboriginal and non-Aboriginal children in Australia’s Northern Territory
Source: PLoS One. 2023 Dec 20;18(12):e0296051. doi: 10.1371/journal.pone.0296051 (PMC10732444; doi:10.1371/journal.pone.0296051)
Supplement: Attachment — Submitted filename: AEDC manuscript_Response to reviewers.docx [file pone.0296051.s002.docx]

| Comments and suggestions | Author’s reply |
| --- | --- |
| Editor’s corrections |  |
| 1. Please ensure that your manuscript meets PLOS ONE's style requirements, including those for file naming. | Checked and revised |
| 2. Thank you for stating in your Funding Statement:  "The study was supported by a grant from the Northern Territory Government through the Child and Youth Development Research Partnership. The funding body was not involved in the study design, data analysis, interpretation of data, or preparation and publication of research papers."  Please provide an amended statement that declares *all* the funding or sources of support (whether external or internal to your organization) received during this study, as detailed online in our guide for authors at http://journals.plos.org/plosone/s/submit-now. Please also include the statement “There was no additional external funding received for this study.” in your updated Funding Statement.  Please include your amended Funding Statement within your cover letter. We will change the online submission form on your behalf. | The Funding Statement has been amended as suggested and included in the cover letter. The statement is now:  “The study was supported by a grant from the Northern Territory Government through the Child and Youth Development Research Partnership. The funding body was not involved in the study design, data analysis, interpretation of data, or preparation and publication of the research paper. There was no additional external funding received for this study.” |
| 3. Thank you for stating the following in the Acknowledgments Section of your manuscript:  "We acknowledge the seven NT Government agencies who participate in the Child Youth and Development Research Partnership (CYDRP) for their sustained support."   We note that you have provided funding information that is not currently declared in your Funding Statement. However, funding information should not appear in the Acknowledgments section or other areas of your manuscript. We will only publish funding information present in the Funding Statement section of the online submission form.  Please remove any funding-related text from the manuscript and let us know how you would like to update your Funding Statement. Currently, your Funding Statement reads as follows:  "The study was supported by a grant from the Northern Territory Government through the Child and Youth Development Research Partnership. The funding body was not involved in the study design, data analysis, interpretation of data, or preparation and publication of research papers."  Please include your amended statements within your cover letter; we will change the online submission form on your behalf. | The Funding Statement has been amended as described above.  The Acknowledgements section recognizes the broader contribution of Northern Territory Government agencies through participation in governance and for approvals to access administrative datasets. The Acknowledgement section also recognizes the Australian Government which funded a national data collection used in the project but did not fund the study.  The revised Acknowledgements section now states:  “The authors acknowledge the support by the NT Government Departments of Health; Education; Territory Families, Housing and Communities; Attorney General and Justice; Chief Minister and Cabinet; Treasury and Finance; and Police, Fire and Emergency Services, through the Child and Youth Development Research Partnership (CYDRP). We also acknowledge the use of data from the Australian Early Development Census (AEDC). The AEDC is funded by the Australian Government Department of Education, Skills and Employment. We thank SA NT DataLink personnel for their technical and administrative assistance in the linkage of datasets and all data custodians for their support with retrieval, preparation and release of the research datasets. The views expressed in this publication are those of the authors and should not be attributed to the government departments who have supplied the data for the study.”  The amended statement has been included with the cover letter. |
| 4. In your Data Availability statement, you have not specified where the minimal data set underlying the results described in your manuscript can be found. PLOS defines a study's minimal data set as the underlying data used to reach the conclusions drawn in the manuscript and any additional data required to replicate the reported study findings in their entirety. All PLOS journals require that the minimal data set be made fully available. For more information about our data policy, please see http://journals.plos.org/plosone/s/data-availability.  Upon re-submitting your revised manuscript, please upload your study’s minimal underlying data set as either Supporting Information files or to a stable, public repository and include the relevant URLs, DOIs, or accession numbers within your revised cover letter. For a list of acceptable repositories, please see http://journals.plos.org/plosone/s/data-availability#loc-recommended-repositories. Any potentially identifying patient information must be fully anonymized.  Important: If there are ethical or legal restrictions to sharing your data publicly, please explain these restrictions in detail. Please see our guidelines for more information on what we consider unacceptable restrictions to publicly sharing data: http://journals.plos.org/plosone/s/data-availability#loc-unacceptable-data-access-restrictions. Note that it is not acceptable for the authors to be the sole named individuals responsible for ensuring data access.  We will update your Data Availability statement to reflect the information you provide in your cover letter. | The data used in the study is sensitive and is held on a secure server with restricted access. The Data Availability statement reflects this restriction.  “The study datasets contain sensitive personal information and are held on a secure cloud-based server with restricted access. Access requires the approval of the ethics committee and data custodians“. The non-author contact information for data access is  [datamanagement@menzies.edu.au](mailto:datamanagement@menzies.edu.au).  The amended statement has been included with the cover letter. |
| 5. Your ethics statement should only appear in the Methods section of your manuscript. If your ethics statement is written in any section besides the Methods, please delete it from any other section. | The ethics statement has been moved from the Declaration section to the Methods section under the sub-heading “Ethics approval” (Page 14, line 299-304). The revised statement is:  “The study was approved by the Human Research Ethics Committee of the NT Department of Health and the Menzies School of Health Research (HREC-2016-2708). A waiver of consent was approved for this study which is based on de-identified population level data. The study was also approved by the First Nations Advisory Group for the CYDRP Program. The group includes independent community members” |
| 6. Please include captions for your Supporting Information files at the end of your manuscript, and update any in-text citations to match accordingly. Please see our Supporting Information guidelines for more information: http://journals.plos.org/plosone/s/supporting-information. | The Supporting Information files at the end of the manuscript have been captioned, and all in-text citations have been matched within the text. |
| Reviewers’ comment | |
| Reviewer 1  This is a generally well-written paper. I have a few comments that need to be addressed: | We thank the Reviewer for her comments. |
| 1. In your Discussion, can you clarify what is novel in your findings? On page 26 you state that much of what you found has been found before - so can you elaborate on what is truly new? | Agreed, we have revised the first paragraph of the Discussion to commence with an additional sentence that reinforces the novelty of the study. (page 25, lines 428-430)  “The study has applied a novel methodological approach to estimate how risk factors combine to predict child development (school readiness), at age five, within a life course conceptual framework.” |
| 2. Abstract - in your Conclusion, which aspects of a child's development need to be captured equally - ie are there dimensions missing which should be highlighted in the abstract? | Agreed, we have revised the Results section of the Abstract to add additional detail. (page 3, lines 47 to 49). |
| 3. On page 5, for ref 50, can you add in the year of the finding that only 41% of Aboriginal children attended pre-school? | Agreed, the year (2021) has been added to the sentence. (Page 5, line 99) |
| 4. Design and Population - when you say you used unit-record level data, does unit= the child? Would be clearer to say this | Agreed, “unit” has been replaced by “individual”. (page 8, line 162) |
| 5. Covariates- in your Discussion, can you say what key covariates you think are missing from the available datasets that should be captured in any future datasets? eg family violence? parenting? quality of preschool? other key determinants of child development? | Agreed, in the Discussion in the sub-section “Main findings” a fifth limitation has been added to describe missing covariates. (page 28, line 501-504) |
| 6. Data analysis - for your logistic regression for non-Aboriginal children, how did you decide on your cut point (I may have missed this)? | Agreed, this information was missing. We have added additional details in the Methods, sub-section ”Data analysis” (page 11, lines 232 to 234) |
| 7. By using CCA approach, you lost a number of children - can you say how those included vs dropped differed? Does this have any implications for the generalisability of your findings? | Within the available datasets there was a high level of missingness for some factors including maternal education, occupation, smoking and drinking and the missingness on these variables was greater for Aboriginal than non-Aboriginal children. Those children with missing data were more likely to be developmentally vulnerable than those included which would bias the estimates towards the null results. To respond to missingness we followed the standard methodological approach. First we tested the assumptions that the missingness was random, and found that it was non-random. We then checked whether the missingness was associated with the outcome after considering other covariates, and we found the chance of being a complete case does depend on the outcome. In this circumstance CCA is the preferred approach, because of the risk of bias associated with use of MI can introduce bias. This information has been highlighted as a limitation in the Discussion section. (page 27, line 489-492) |
| 8. On page 29, you discuss the need for "integrated or seamless preschool and early childhood care and education programs" ...easy to say but hard to do - can you give any examples of successful programs? | Agreed, In the Discussion section, in the sub-section Place-based strategies, we have added examples of long- standing integrated services in Australia (page 30, line 523 to 529) |
| Reviewer #2  Thank you for the opportunity to read this manuscript. Clearly a lot of work went into it and tackles an important issue – Aboriginal child development, an area of great importance to Aboriginal communities. Large sections of the paper are very well-written. | We thank the reviewer for the comments. |
| 1. However, I have several major concerns. I struggled to hear the voices of Aboriginal communities and researchers in this work (apologies to any of the authors who are Aboriginal and/or Torres Strait Islander). In recent years, a number of eminent Aboriginal researchers have tried to guide research in a new direction and I think this manuscript would be strengthened by following some of the recommendations of those researchers (e.g. Maggie Walter, Stephen Harfield’s paper Assessing the quality of health research from an Indigenous perspective: the Aboriginal and Torres Strait Islander quality appraisal tool). In particular, this paper would benefit from being written from a strengths-based perspective. | The authors acknowledge the sensitivities and risks for research involving Aboriginal people and are familiar with the recent guidance on working with Aboriginal people including the work by Hartfield and the NHMRC guidelines - *Ethical conduct in research with Aboriginal and Torres Strait Islander*  *Peoples and communities: Guidelines for researchers and stakeholders (2018).*  This project fits within a broader body of research – the Child and Youth Development Research Partnership (CYDRP) which has the specific aim of improving services and outcomes for NT children and young people. Most projects report information for Aboriginal children, who are more than 40% of NT children. The program engages with Aboriginal people at multiple levels.   - The research is conducted with the approval of a Human Research Ethics Committee, which requires responses by researchers against the NHMRC guidelines. The HREC review includes review by an Aboriginal research committee who provide advice on projects and have the right of veto for projects. - All projects within the CYDRP program require approval and ongoing progress reports to the First Nations Advisory Group, who provide guidance on both the relevance and cultural safety of the projects. The advisory group also contributes to the research agenda for the program. - Many projects have Aboriginal investigators, although there were no Aboriginal investigators in this project. - The results of studies are widely distributed – through scientific papers, research briefs and by presentations, including to relevant Aboriginal service agencies.     This study was designed to inform responses to the Australian Governments’ “Closing the Gap Strategy”. The current strategy was developed as a partnership with Aboriginal organisations and contains approved targets. These targets include the standard measurement of AEDC domains used in this study, which are based on developmental vulnerability. |
| 2. Also, Aboriginal should always have a capital ‘A’ and this needs to be corrected in the supplementary material. Similarly, the noun “Aboriginals” could be replaced in the supplementary material with something like “Aboriginal people”. | Agreed, we apologise for incomplete editing in the supplementary material. |
| 3. I felt there was sometimes a blurring of the distinct concepts of child development, readiness for a school system that was not set up for Aboriginal children, and AEDC scores. I think the manuscript could benefit from being careful to articulate which concept is being referred to throughout. | We acknowledge the school system is a western construct and was not developed to respond to Aboriginal learning styles nor is it consistent with Aboriginal concepts of child development. The debate about the applicability of AEDC as a measure of child development for Aboriginal children is summarized in the Background (pages 6-7, lines 124-147). Including the argument that the AEDC instrument measures “school readiness” rather than “child development”, particularly for Aboriginal children.  We have written the manuscript to accommodate the duality between the widespread use of AEDC as a measure of “child development” (as used in the Closing the Gap Target of “developmentally on track”) or as a measure of “school readiness” which is more appropriate for Aboriginal children. The title of the manuscript reflects this duality.  We have reviewed our use of “child development” and “school readiness” in the manuscript and for consistency have replaced the terms in various places with “child development (school readiness)”.  The changes have been made at   - In the Abstract (page 2, line 30) - Background (page 7, line 147) - Methods, (page 9, lines 181 and 195), (page 12, line 242) - Discussion (page 25, line 428), page 25, line 434), page 26, lines 453 and 464), page 27, line 488). - Conclusion (page 30, line 550) |
| 4, Could the authors provide more detail about why the AEDC scores were measured formatively – I would have thought that the construct (child development) informs the indicators (AEDC scores), rather than the other way around, so a more detailed explanation or diagram would help (I note that Fig 1 has been included, but I assume that the correct reading of Fig 1 is that ‘true’ physical health and well-being informs ‘true’ child development and the AEDC score for physical health is not in this figure, but if it was, there would be an arrow from ‘true’ physical health and well-being to AEDC score). | The reason for using formative assessment of indicators has been highlighted in the Data analysis section (page 12, lines 258 to 260). We provide further explanation below.  The main issue for the approach to the analysis is how the AEDC measure is conceptualised and whether it is a combination of its five domains (formative) or a concept of “child development” for which the five domains reflect elements of the central construct (reflective).  In a reflective model, the latent construct exists (in an absolute sense) independent of the measures (Borsboom et al., 2004, Rossiter, 2002). Typical examples of reflective scenarios include measures of attitudes and personality. In contrast, in a formative model, the latent construct depends on a constructivist, operationalist, or instrumentalist interpretation. (Borsboom et al., 2003). For example, the human development index (HDI) does not exist as an independent entity. Rather, it is a composite measure of human development that includes health, education, and income (UNDP, 2006). In this case, any change in one or more of these components is likely to cause a change in a country's HDI score.  We believe the AEDC concept of child development shares the same theoretical principles as the HDI where a change in one or more domains changes the overall child development result and have applied a analytic method appropriate for this construct.  The reviewer is correct in the interpretation of Figure 1, which includes the domain of physical health and wellbeing with an arrow to the construct of “Early child development outcomes” as measured by the AEDC score. |
| 5. Underlying the discussion appears to be an assumption that Aboriginal children should be prepared to a school system set up largely by non-Aboriginal people. I note that many Aboriginal families would like the system to fit to their children’s needs rather than the other way round. | We acknowledge this assumption, which reflects the broader community expectation that all Australian children participate in the formal Australian education system. This expectation is consistent with the Closing the Gap Strategy and associated targets. The authors recognize that Aboriginal communities may have different expectations and other systems of education and learning but this discussion is beyond the scope of this study. |
| 6. The results from the complete case analysis (CCA), indicator variable for missing data approach and MICE (Supplementary Table 1) were very different – some variables were significant CCA but not the other approaches and some coefficients changed direction. Given this, how would their conclusions have changed if MICE was the preferred approach? | The analysis was based on the standard statistical recommendation that if missingness is non-random and if the missingness is associated with the outcome, after adjusting for other covariates, then CCA is the appropriate method. Using MICE for the analysis would bias the estimates towards the null and is statistically inappropriate. See also our response to Reviewer #1, Comment 7 and to the next response to an error in the text (Reviewer #2, comment 7) . |
| 7. I am curious about the finding that missingness did not depend on the outcome. Looking at Table 1, missingness was much more common among Aboriginal children and AEDC scores were lower for Aboriginal children, so I would have expected an association between missingness and AEDC scores. It would be interesting to see data on AEDC scores for children missing data vs AEDC scores for children not missing data. How did the authors conclude missingness did not depend on the outcome? | We apologise for this error in drafting. Missingness in the covariates was related to the outcome variable. Children who missed observation on these variables were more likely to be developmentally vulnerable in one or two domains. So, missingness was not random and Multiple Imputation would introduce bias. CCA is the appropriate method. We have corrected this error in the text. (page 14, lines 288-292) |
| 8. Some of the data analysis section is hard to follow and could be rewritten and restructured so it is clearer what the aim of each step is and so that information flows in a logical order (e.g. “in-sample” appears at line 254, but “in-sample” and “out-of-sample” is not explained until later. | Agreed, we have added an explanation of their meaning in the revised manuscript. (page 12 lines 258-259 and page 13, line 264)). In-sample and out-of-sample are standard terms in predictive model studies.  More generally we have edited the Methods/Data analysis section for readability (pages 11-13, with edits in various lines between 219-280) |
| 9. The decision-making about how the final models were selected is difficult to follow (e.g. line 324 – it is not clear how the authors ended up with 17 competing models and how they went from those 17 to 4). There is mention of the supplementary material, but there appear to only be 15 models in the supplementary material and it’s not clear what the 4 final models are, nor how the 2 presented in the main paper were chosen. Perhaps a diagram could explain this. | For clarity, we have edited the description of the selection process, which reduced the 17 models down to a final 2, based on model assessment of predictive fit, power, utility and simplicity (page 20, lines 340-343). The details of the selection analysis are provided in the Supplementary information |
| 10, Line 121: Suggest rewriting the first sentence as not currently clear. However, the rest of this paragraph is very good and perhaps the ideas in this paragraph could inform the rest of the paper a bit more. | Agreed, the sentence has been revised (page 6, lines 124-125)  As discussed in response to Reviewer #2, comment 3 we have edited the manuscript to the general use of child development (school readiness). |
| 11. Line 182: Typo | Agreed, and corrected (page 9, line 184) |
| 12. Line 200: Specify the variables more exactly (e.g. what were the categories of education and employment?) | The variables are listed in the descriptive tables, and our preference is not to duplicate the extended list in the body of the text. |
| 13. Line 223: Explain what total AEDC summary scale scores are | Agreed, additional description has been added (page 11, line 223) |
| 14. Line 229 and 230: What are these models? What is the binary outcome for the non-Aboriginal children? | Agreed, additional detail has been added as response to Reviewer 1, Comment 6 (page 11, lines 230-234) |
| 15. Line 238: Include how many children were excluded and on what grounds. | Children were not excluded prior to the development of the predictive models which were based on complete cases analysis. Each of the 17 models have varying numbers of children depending on the availability of data for the variables included in the model.  In the manuscript, we have added the number of Aboriginal children (606) and non-Aboriginal children (738) available in the final two models. (Page 20, line 344 and page 21, line 362) |
| 16. Line 323: What does “Added percentile of AEDC domains” mean? Need to clarify that p-values at the end of Table 1 are for comparisons of Aboriginal and non-Aboriginal children while p-values for the rest of the table for comparisons of AEDC values and the variable. | We have revised the description in the text in Table 2 for clarity, (page 19, final two lines of Table 2)  We have edited the Note for Table 1 and Table 2, for clarity, and added an additional note in Table 2 (Table 1-page 17, lines 321-322 and Table 2- page 19, lines 337-338). |
