# Supplementary material for: Predicting child development and school readiness, at age 5, for Aboriginal and non-Aboriginal children in Australia’s Northern Territory
Source: PLoS One. 2023 Dec 20;18(12):e0296051. doi: 10.1371/journal.pone.0296051 (PMC10732444; doi:10.1371/journal.pone.0296051)
Supplement: Attachment — Submitted filename: Rebuttal_4052023.docx [file pone.0296051.s003.docx]

| Comments and suggestions | Author’s reply |
| --- | --- |
| Reviewer #2: |  |
| I don't believe the authors have adequately addressed my comment re missing data, or Reviewer 1's comment, and in the process of re-reading it, I realised the authors may not have accurately described the recommendations laid out in the two papers on missing data that they referenced.  It was not clear from what they wrote whether the authors thought their data were missing at random (MAR) or missing not at random (MNAR). They wrote “Missingness in the covariates was related to the outcome variable. Children who missed observation on these variables were more likely to be developmentally vulnerable in one or two domains. So, missingness was not random and Multiple Imputation would introduce bias. CCA is the appropriate method.” Having missingness in the covariates related to the outcome variable, does not (of itself) distinguish between MAR (which, despite its name, is not completely random) and MNAR and there is no 'test' to distinguish between MAR and MNAR. Given that it is hard to know whether data is MAR or MNAR, it is also not always clear whether CCA or MI is preferable and often both are used and the results compared.  Despite this, the authors claim that CCA is preferable, “In this case, methodological studies have reported that multiple imputation (MI) tends to be biased away from the null and complete case analysis (CCA) is a better approach (83, 84)”, I note that reference 84 actually starts the Discussion section with “Our comparison of MAR-based MI with CC analysis for regression models with one or two incomplete covariates shows no universally consistent advantage of one method over the other, although MI appears to be superior across a wider range of settings.“ For reference 83, despite the title “Accounting for missing data in statistical analyses: multiple imputation is not always the answer”, the authors themselves chose MI for the analysis they were interested in.  Given all of this, the approach of doing both CCA and MI in this study was welcome, but the results from both approaches should be discussed. Re-reading my comment, I realise I could have made this point more clearly. The case is better made in Sterne JA, White IR, Carlin JB, Spratt M, Royston P, Kenward MG, Wood AM, Carpenter JR. Multiple imputation for missing data in epidemiological and clinical research: potential and pitfalls. Bmj. “Where complete cases and multiple imputation analyses give different results, the analyst should attempt to understand why, and this should be reported in publications.”  The sentence “In this case, methodological studies have reported that multiple imputation (MI) tends to be biased away from the null and complete case analysis (CCA) is a better approach (83, 84)” needs to be re-written to better reflect the content of the papers they are referencing. | We note the reviewer’s concern.  For clarity, we have edited the section on Missing data handling, including the sentence.  “The missing mechanisms is potentially missing not at random (MNAR) as the reason for missingness was explained by other missing covariates and the missing values themselves might be more likely to correlate with the missing values (for example a woman with low level of education is more likely to escape the question because of social desirability issues).” (line 287 to 291)  In the section we also describe sensitivity testing, using two methods for missing handling data, which demonstrate different covariates in the final model to those identified with CCA, which further supports the use of CCA. (Page 14 lines 286 to 294)  In relation to testing whether missingness is MAR or MNAR, we agree there are no statistical tests to identify the type of missing mechanisms however there are ways to differentiate between them with some level of certainty, as outlined by Enders (Enders CK. Applied missing data analysis. Guilford Publications; 2022 Jul 1.)  We disagree with the suggestion by Reviewer 2 that we may have misinterpreted the cited references by stating “*If the missingness mechanism fulfils the criteria of MNAR and the missingness is not dependent on the outcome, both methodological studies reported that using MI biases the result towards the null and CC is a better approach”. We copy* the abstract sections of the two papers:  i) The Hughes et al paper states “*For most regression models, CCA gives unbiased results when the chance of being a complete case does not depend on the outcome after taking the covariates into consideration, which includes situations where data are missing not at random.” (*Hughes et al., 2019); and,  (ii) The White et al article also states “*When data are missing completely at random, both methods have negligible bias, and MI is more efficient than CC across a wide range of scenarios. For other missing data mechanisms, bias arises in one or both methods. In our simulation setting, CC is biased towards the null when data are missing at random. However, when missingness is independent of the outcome given the covariates, CC has negligible bias and MI is biased away from the null”.* (White and Carlin, 2010)*.*  As a further sensitivity analysis, we presented results from both analyses. (Supplementary information). The most important thing that we need the reviewer should consider is that: (i) The purpose of the first analysis was for variable reduction. To identify covariates associated with the outcome, we used a cut-off up to a p-value of 0.1, which we believe minimizes the risk of important covariates being missed and not included in the predictive model;  (ii) The prediction model fitting involved multiple stages and included the assessment of the covariates which were found most prominent in the MI and MICE models. This further minimised the risk of excluding important covariates for prediction;  (iii) We would also expect relatively similar results had we used MI as we followed a standard statistical recommendation; and  (iv) There were 17 potential predictive models developed at the first stage of analysis but after further re-analysis and using fit criteria and simplicity, we selected two models for interpretation. We believe the triangulation provided by these multiple methods has minimised the potential risk of bias associated with missingness.  Thanks,  We have revised the section to improve clarity however the content remains consistent with the recommendation of the cited references. |
| Reviewer #3 |  |
| Given this paper has already been reviewed by others, I have focused my attention on the response of the authors to the original reviewer comments—although I have a few additional (minor) comments on other aspects of the paper. Overall, I thought the paper was well written, included a thorough approach to the analysis and is centred on an important topic in research, policy and practice. | We thank the reviewer for the comments |
| My main area of concern was highlighted by Reviewer 1 (point 6): and concerned the approach to dealing with data missing not at random. The authors response suggests that a CCA is preferred to a multiple imputation approach. This may be the case but I am not an expert in these methodological issues – my readings suggest, on balance, that this is a fair argument. However, it remains the fact that around 52-60% of the Aboriginal and non-Aboriginal sample were included in the final prediction models. Given this scale of missingness and likelihood of bias, this appears to me to be a major issue in terms of the impact on the selection of variables and estimated effects. | As described in our response to Reviewer 2, above, we would expect similar relatively the same results for CCA, and had we used MI if data was missing at random, however as demonstrated in the sensitivity analysis, this was not the case and CCA is the preferred method.  In relation to the sample, the size of the sample remains efficient for developing the predictive model as we followed a standard statistical recommendation. Furthermore, the sample is still efficient to develop a valid prediction model using SmartPLS software. |
| Perhaps part of the issue here is the (from what I can tell) reliance on Model I results in informing the selection of variables in the prediction models – there appears to be a significantly smaller sample for the CCA analysis compared with Models II and III. In any case, all of this, of course, places limitations on the implications of the final model results for policy and practice—and while the issues of bias are mentioned as a limitation, I’m not sure the authors do enough to discuss this concern or asses the likelihood of it not being serious. | Noted  The PLS-SEM model has been developed to be efficient in a small sample-size scenario such as the one we have used (Shmueli 2019). We also used various model triangulation approaches by developing several potential candidate prediction models (17 models) before selecting the final prediction model using standard criteria. We believe this approach has minimized the risk of missing other alternative prediction models. The only issue of concern is the potential for misclassification bias that might happen between the included and excluded samples, which we highlighted as a limitation in the discussion section. |
| - The terms ‘positive’ and ‘poorer’ outcomes in the Abstract should be defined or a more specific term used.  It also wasn’t clear to me what was meant by the term ‘not equally captured’ | Thanks,  Agreed,  We have replaced the word “positive” with “better” outcomes in the abstract and other sections where this term is used. (Abstract line 37 and 45)  Noted,  The Australian Early Development Census uses measures across five domains to assess the overall development of a child. Each domain reflects a different aspect of child development, and the measurement of each domain should contribute equally to the final result of the assessment. The analysis in our study demonstrates that the results for the five domains are not contributing equally to the AEDC results.  This information has been presented in the Result and Discussion sections. (Line 356 to 360 result section and 435 to 441 in the discussion section) |
| - I would have preferred the Conclusion of the Abstract to tell us what factors are important, based on the model results | Agreed,  We have added a sentence in the Conclusion to make a more direct reference to the multiple factors influencing development. The factors are reported in the Results of the Abstract, so we have not repeated them in the Conclusion. |
| - First para of page 5: assume this relates to all Australian children (not just NT)? | Agreed, and revised (Page 5 line 122) |
| - Page 15, line 319: should clarify that the univariable association is with the AEDC total score (although the effect is not reported; instead it reflects that there was a significant association); also, from what I can tell, these are associations in the regression models of step 1 as opposed to the predictive model? If I have interpreted this correctly then a few tweaks need to be made to the text and the table titles that follow | Agreed,  This reference is to a crude demonstration of the presence of an association between the AEDC total score and the named complications during pregnancy (Table 1). For clarity, we have revised the text. See lines 309 to 310 and Title of Table 1 lines 321 to 322. |
| - Page 30: the inclusion of more details on place-based strategies is very useful. Is there any information on whether these have been evaluated and therefore effective? | Agreed,  Though place-based strategies had a long history in Australia, we could not find comprehensive output-based effectiveness studies and only a few reports of general aspects of the strategies.  We have updated the ‘place-based strategy’ section of the Discussion with reference to these studies and mentioned the gap in more comprehensive evaluations. (Lines 517 to 539). |
| - Page 31 (Conclusion): I don’t disagree that SES and preschool attendance are implicated in structural inequalities, however, I’m not sure that the presence of these factors in the model for Aboriginal children suggests this given it is a within-Aboriginal sample model. | Agreed,  We have amended the sentence replacing describing the predictors for Aboriginal children by removing “systemic” and replacing “in” with “within” to reinforce that the differences demonstrated in the analysis are within the Aboriginal sample. |
