# Supplementary material for: Predicting child development and school readiness, at age 5, for Aboriginal and non-Aboriginal children in Australia’s Northern Territory
Source: PLoS One. 2023 Dec 20;18(12):e0296051. doi: 10.1371/journal.pone.0296051 (PMC10732444; doi:10.1371/journal.pone.0296051)
Supplement: Attachment — Submitted filename: Rebuttal_22112023.docx [file pone.0296051.s004.docx]

| Comments and suggestions | Author’s reply |
| --- | --- |
| Reviewer #2: |  |
| Reviewer #2: The authors have undertaken more work examining the missingness in the data. This version states, “Furthermore, we also checked that the missingness was not dependent on the outcome (AEDC) after adjusting for the other covariates”. It is not clear how this was checked and there has a confusing history about the nature of the missing data.  In the first draft, the authors stated that missingness was not dependent on the outcome. When this was queried, based on results in Table 1, the authors advised that this was an error and revised the text to, “missingness was associated with the outcome (AEDC) i.e., children who missed observation on these variables were more likely to developmentally vulnerable on one or two domains”. In this version, they state that missingness is not dependent on the outcome after adjusting for the other covariates.  Unfortunately, the authors did not provide evidence to support this new statement, nor a description of the methodology used. If there is no association between missingness and the outcome, conditional on the other covariates, I accept that the references used are appropriate. If not, my recommendation would remain that:  - No new analysis is required. Only some revision of the written material is required.  - Multiple approaches are appropriate  - Differences in results between the complete case analysis and the 2 sensitivity analyses should be discussed  - The wording around those references should be changed. | Thank you, reviewer, for your dedication to improve the strength and clarity of our work.  The new version of our manuscript included how we checked that the missingness was not dependent on the outcome variable after adjusting for other covariates—and reads as “We checked this by first creating a missing indicator variable for each variable with missingness (yes=1, no) and then fitting regression to model the missingness accounting for other covariates.”  I hope this clarifies how we checked this. |
